# The Effect of Weekly 50,000 IU Vitamin D_3_ Supplements on the Serum Levels of Selected Cytokines Involved in Cytokine Storm: A Randomized Clinical Trial in Adults with Vitamin D Deficiency

**DOI:** 10.3390/nu15051188

**Published:** 2023-02-27

**Authors:** Dana A. Bader, Anas Abed, Beisan A. Mohammad, Ahmad Aljaberi, Ahmad Sundookah, Maha Habash, Ahmad R. Alsayed, Mohammad Abusamak, Sami Al-Shakhshir, Mahmoud Abu-Samak

**Affiliations:** 1 Department of Clinical Pharmacy and Therapeutics, Applied Science Private University, Amman 11931, Jordan; 2 Pharmacological and Diagnostic Research Centre, Faculty of Pharmacy, Al-Ahliyya Amman University, Amman 11931, Jordan; 3 Department of Pharmaceutical Sciences, Fakeeh College for Medical Sciences, Jeddah 21461, Saudi Arabia; 4 Department of Pharmaceutical Sciences and Pharmaceutics, Applied Science Private University, Amman 11931, Jordan; 5 Department of Clinical Nutrition and Dietetics, Applied Science Private University, Amman 11931, Jordan; 6 Michael Sayegh, Faculty of Pharmacy, Aqaba University of Technology, Aqaba 77110, Jordan; 7 Department Surgery, School of Medicine, Al-Balqa Applied University, As-Salt 19117, Jordan; 8 Amman Eye Clinic, Amman 11931, Jordan

**Keywords:** vitamin D deficiency, vitamin D_3_, cytokine, storm, interleukin 6, interleukin 10, interleukin 1β, TNF-α

## Abstract

This research aimed to evaluate the effects of high-dose cholecalciferol (VD_3_) supplements (50,000 IU/week) on selected circulating cytokines associated with cytokine storms in adults with vitamin D deficiency. This clinical trial, based in Jordan, included 50 participants receiving vitamin D_3_ supplements (50,000 IU/week) for 8 weeks; the exact number was assigned to the control group. Interleukin-6 (IL-6), interleukin-1β (IL-1β), interleukin-10 (IL-10), tumor necrotic factor-α (TNF-α), and leptin were measured in serum at baseline and 10 weeks (wash out: 2 weeks). Our results revealed that vitamin D_3_ supplementation significantly increased the serum levels of 25OHD, IL-6, IL-10, IL-1β, and leptin compared with baseline. In contrast, the serum level of TNF-α insignificantly increased in the group receiving vitamin D_3_ supplementation. Although the observations of this trial may refer to a potential negative effect of VD_3_ supplementation during cytokine storms, further trials are required to clarify the potential benefits of VD_3_ supplement during cytokine storms.

## 1. Introduction

The new coronavirus infection (COVID-19) is a global pandemic that has aggressively propagated worldwide [1]. Severe acute respiratory syndrome-coronavirus-2 (SARS-CoV-2) is responsible for the pandemic viral pneumonia known as COVID-19. A recent study observed elevated serum levels of specific cytokines, such as those seen during the COVID-19 cytokine storm (CS), which may be associated with severe complications resulting from the infection [2]. These cytokines include tumor necrosis factor-alpha (TNF-α), interleukin-1 beta (IL-1β), IL-6, IL-, 10, IL-17, interferon-gamma (IFN-γ), and many other cytokines [1]. Accordingly, hypercytokinemia has been recently suggested to be one of the main hallmarks of COVID-19 [1]. However, many COVID-19 symptoms can be treated based on the patient’s clinical condition. Hence, because there is no specific treatment for COVID-19 yet, supportive care, including dietary vitamins and minerals for infected persons, can be highly effective.

In this manner, vitamins D (VD) and C, in addition to zinc supplementations, are recommended by the Jordan Ministry of Health as a part of the treatment protocol for COVID-19 patients. VD increases innate cellular immunity and provides direct antibacterial activity against various microorganisms, including enveloped and nonenveloped viruses [3]. It has also been shown that VD may mitigate the CS induced by the innate immune system [4]. A previous study [5] concluded that VD modulates the immune response via its effects on dendritic cells (DCs) and T cells. This may enhance the clearance of the virus and decrease inflammatory reactions associated with symptoms. However, vitamin D deficiency (VDD) is still a global problem; over one billion people are either VD deficient or insufficient, and the incidence of VDD was reported to be higher in Mediterranean countries such as Jordan [6,7,8]. VDD is known to be directly associated with bone disorders, but nonskeletal outcomes grabbed the most attention [9]. It was linked with different disorders, including diabetes, cardiovascular disease (CVD), atherosclerosis, and cancer [10,11], and certain abnormal immune conditions, including infections [5]. Recent reviews indicated the ability of VD to reduce the risk of microbial infections through different potential mechanisms: physical barrier, natural cellular, and adaptive immunity [12,13].

Consequently, raising 25-hydroxyvitamin D (25OHD) levels by 1,25(OH)2D3 (VD_3_) supplementation is highly recommended to reduce the risk of infection and is advised as part of the treatment protocol for people who are sick with influenza [4]. However, the modulatory effects of VD on proinflammatory and anti-inflammatory, as well as cellular and humoral immune, responses are mixed and unclear. There is a study that showed that VD could suppress the production of T-helper (Th)1 proinflammatory cytokine [14] and augment Th2 cell development [15]. Furthermore, VD_3_ enhances T regulatory cell induction, thus inhibiting inflammatory processes [16].

A previous study [17] showed the safety of VD and a protection activity against acute respiratory tract infection. VDD has been correlated with acute respiratory distress syndrome (ARDS), and case fatality rates (CFRs) increase with age and comorbidity with chronic diseases, both of which are associated with lower 25OHD concentration [4]. Therefore, this randomized clinical trial (RCT) was designed to measure serum levels of IL-1β, IL-6, IL-10, and TNF-α as part of the immune response during CS before and 8 weeks after high-dose VD_3_ 50,000 IU in adults with VDD.

## 2. Materials and Methods

### 2.1. Patient Characteristics

This RCT was approved by the Institutional Review Board of Applied Science Private University (ASU) (protocol number 2020-PHA-16) and undertaken between October 2020 and December 2020. The clinical trial was conducted following the Helsinki Declaration. Each individual who was enrolled provided informed consent for this clinical trial. With an average baseline age of 38.37 ± 9.77 years, volunteers included were Jordanian and from ASU staff and their families (ranging from 30 to 66). Eligible participants were included in the trial depending on a diagnosis of VDD confirmed by medical consultants at Ibn Al-Haytham clinical laboratories. Because prolonged VD3 administration is related to the formation of kidney stones, patients with kidney abnormalities were excluded from the study [18]. COVID-19 or chronic medical conditions, such as osteoporosis, cancer, endocrine disorders, and a history of allergic responses to VD3 supplements, were also among the exclusion criteria from this study.

### 2.2. Intervention

**Figure 1:**
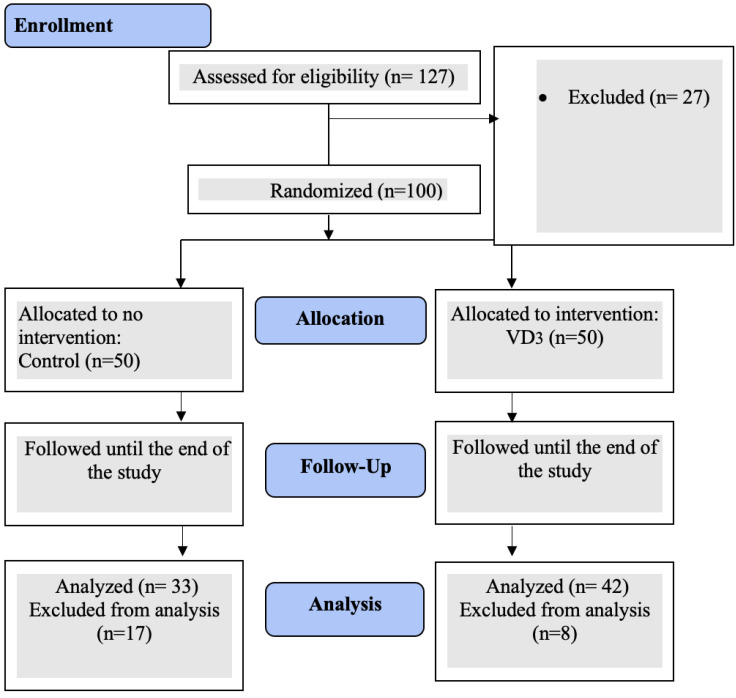
Consort flow diagram for the study.

Before and after the VD3 supplement, baseline and follow-up values of anthropometric and clinical parameters were collected. At the conclusion of the 8-week interventional phase, the subjects underwent a 2-week washout phase before and following VD3 administration. VD3 is a fat-soluble vitamin with a long half-life; a washout period was achieved to avoid the potential effect of its cumulative dose. Then, all participants’ follow-up measurements were obtained. An independent statistician developed a computer-generated randomization process. According to the consortium chart (Figure 1), hundreds of eligible participants were divided into two groups: group 1 received once weekly 50,000 IU of VD3 in a Hi-Dee soft gelatin capsule (United Pharmaceuticals Company, Amman, Jordan). Participants in group 2 did not receive any supplementation and acted as the control group. In compliance with the Endocrine Society’s clinical guidelines for treating VDD in adults, therapeutic protocols for VD3 supplements were approved [19]. Similarly, administering VD3 to individuals throughout 12 months produced no toxicity [19]. All participants’ adherence to the therapy protocol was monitored by periodic text messages sent to their mobile phones.

### 2.3. Anthropometric Measurement

This RCT was conducted throughout the winter of 2020 at the ASU Pharmacy school laboratories to minimize seasonal fluctuations in vitamin D assays in the blood [20]. At the beginning and end of the experiment, anthropometric measurements, such as body mass index (BMI), body weight (BW), hip (H) circumference, waist/hip ratio (WHR), height (Ht), and waist (W) circumference, were recorded.

### 2.4. Clinical Parameter Assays

Serum assay of clinical parameters was collected into labeled Eppendorf tubes at Ibn Al-Haytham Hospital, Clinical Laboratories Department, Jordan.

The chemiluminescence immunoassay LIAISON 25OHD assay (DiaSorin, Saluggia, Italy) measured total serum 25OHD. The assay quantifies serum 25OHD and is cross-reactive with 25OHD2 and 25OHD3. Its lower limit was 4 ng/mL. An enzyme immunoassay kit measured serum leptin levels (leptin EIA-5302, DRG Diagnostics, Marburg, Germany). Test sensitivity was 0.1 ng/mL. An enzyme immunoassay kit tested serum PTH levels (PTH Intact EIA-3645, DRG Diagnostics, Marburg, Germany). The sensitivity was 1.57 pg/mL. The calcium-ARSENAZO kit (M11570i-15) and the phosphorus phosphomolybdate/Uv kit (M11508i-18, BioSystems, Barcelona, Spain) were used to measure the levels of calcium and phosphorus (PO4) in serum. Serum IL-6 concentration was measured using the Human ELISA KIT (ab178013, Abcam, Newark, NJ, USA). Using a Human ELISA Kit, serum IL-10 was measured (ab185986, Abcam). Human ELISA KIT assessed serum IL-1β (ab214025, Abcam). The Human ELISA KIT assay measured serum TNF-α (ab181421, Abcam).

### 2.5. Statistical Analysis

SPSS version 27 for Windows was used to execute the statistical analysis. A paired *t*-test was performed to determine any significant variations in each trial group before and after the delivery of the VD3 supplementation. Two independent sample *t*-tests were utilized to identify whether there were significant differences between all items of distinct groups (control, D_3_). Using correlation analysis, correlations were investigated between the serum levels of TNF, IL-1β, IL-6, IL-10, and 25-OHD, as well as between their ratios (TNF-α /IL-10, IL-1β /IL-10, and IL-6/IL-10). Simple linear regression was used to investigate the effect of VD_3_ supplementation on the items (TNF-α, IL-1β, IL6, IL10, ratio (TNF-α /IL-10), ratio (IL-1β/IL-10), ratio (IL-6/IL-10)), while multiple linear regression analysis was used to determine the predictors of the items (TNF-α, IL-1β, IL-6, IL-10, ratio (TNF-α /IL-10), ratio (IL-1β/IL-10), and ratio (IL-6/IL-10)) at the follow-up level for the (D) group. The Kolmogorov–Smirnov test was utilized to test the normality of distribution for laboratory measurements. The results displayed a normal distribution curve.

## 3. Results

### 3.1. Baseline Values of the Participants

A total of 75 out of 127 (59.1%) participants in the trial adhered to the protocol and finished the intervention period that lasted for 8 weeks. As indicated in Figure 1, the reasons why participants dropped out (*n* = 27) included noncompliance, not fulfilling inclusion criteria, and dropping out from the intervention group (*n* = 8) and the control group (*n* = 17). In this trial, 50.7% were female, and 49.2% were male. Morning sun exposure was practiced by 57.3% of participants. Other baseline percentages and frequencies of analyzed anthropometric and lifestyle variables are displayed in Table 1.

Participants’ average age was (38.37 9.77). When measured at baseline, all other serum markers had mean values that were within normal limits. Descriptive analysis for anthropometric parameters, including BMI (27.90 ± 4.76), waist and hip circumferences, and WHR, are shown in Table 2.

### 3.2. Baseline Clinical Characteristics

The baseline mean value for serum 25OHD was (17.29 ± 6.18) ng/mL (all participants were VD deficient). None of the participants presented in this trial with a baseline serum 25OHD level equal to or greater than 30 ng/mL. All participants’ baseline values of all serum parameters, including PTH, Ca, and PO_4_, were within normal ranges. A descriptive analysis of the clinical parameters is presented in Table 3. Table 4 shows baseline mean values for the serum levels of IL-1β, IL6, IL10, and TNF-α and their ratios.

### 3.3. Connection between Selected Cytokine Variables and 25OHD Concentrations

In this trial, selected proinflammatory cytokines (IL-1β, TNF-α, and IL-6) and anti-inflammatory cytokine (IL-10) showed statistically significant intercorrelations (Table 5). At baseline, serum 25OHD levels showed a significant inverse correlation with serum IL-1β levels (R= −0.280, *p* = 0.015). Serum IL-1β levels also showed significant positive correlations with IL-6 (R = 0.236, *p*= 0.041) and IL-10 (R = 0.239, *p* = 0.039). Pearson correlation analysis showed no correlation between serum IL-6 and IL-10 levels. Other baseline intercorrelations between studied cytokines ratios are listed in Table 5.

The Pearson correlation analysis showed no intercorrelation between serum cytokine levels in the VD3 group, as shown in Table 5. Serum 25OHD levels showed a weak but insignificant positive correlation with serum IL-1β levels. However, this trial has shown a significant reverse correlation between proinflammatory cytokines and anti-inflammatory cytokines. Table 5 shows the correlation between each cytokine and 25OHD level.

### 3.4. Changes in the Serum Levels of 25OHD and PTH

Paired sample *t*-tests showed a significant difference in the follow-up mean 25OHD and PTH levels among participants of the D_3_ group (41.39 ± 12.19 vs. 16.41 ± 4.99 and 16.69 ± 8.72 vs. 37.88 ± 6.82, P^A^ < 0.001, respectively). Independent sample *t*-tests determined significant differences in 25OHD and PTH levels between the control and D_3_ groups. There were significant differences in serum 25OHD and PTH between the control and D_3_ group at follow-up (17.31± 6.74 vs. 41.39 ± 12.19 and 33.85 ± 10.62 vs. 16.69 ± 8.72, P^C^ < 0.001, respectively, P^C^ < 0.001), as shown in Table 6.

### 3.5. Changes in the Serum Levels of Selected Cytokines Associated with Cytokine Storm at Baseline and 10-Week Follow-Up

At the end of this study, a paired *t*-test showed a significant difference in mean IL-1β, IL-6, and IL-10 levels. Mean IL-1β significantly increased with a change to 4.41 ng/mL (7.63 ± 2.36 vs. 3.22 ± 0.99, P^A^ < 0.001) in the D_3_ group. The application of the statistically independent *t*-test showed a significant difference in mean IL-1β between D_3_ and the control group (3.59 ± 2.71 vs. 7.63 ± 2.36, P^C^ < 0.001). There was a significant change between the mean IL-6 at baseline and follow-up among those in the D_3_ group (5.5 ± 6.51 vs. 26.99 ± 14.47, P^C^ < 0.001). At the 10-week follow-up, mean IL-10 levels were significantly increased with a change to 2.45 ng/mL (2.01 ± 0.59 vs. 4.46 ± 4.67, P^A^ = 0.001) in the D_3_ group. IL-10 levels were significantly higher in the D_3_ group compared with the control group, 4.46 ± 4.67 and 2.39 ± 1.39, respectively, with a *p*-value of P^C^ = 0.016. Table 7 presents the baseline results and follow-up changes of the clinical variables studied in this trial.

### 3.6. Stepwise Regression Analysis

The multivariate stepwise regression analysis revealed significant mediating factors (IDVs) on the circulatory levels of selected cytokines associated with CS at the 10-week follow-up supplementation of VD_3_ 50,000 IU once a week. TNF levels were only mediated by age factor (R = 0.413, R^2^ = 0.170, *p* = 0.007). Changes in IL-1 level values observed in the VD_3_ interventional group were significantly mediated by body weight (R = 0.311, R^2^ = 0.097, *p* = 0.045).

Regarding the TNF/IL-10 ratio, WHR only was selected by the stepwise regression model among all IVDs to be involved in the positive relationship between elevated 25OHD levels and the TNF/IL-10 ratio (R = 0.348, R^2^ = 0.121, *p* = 0.024), as observed in Table 8.

## 4. Discussion

At the end of the trial, high doses of VD_3_ supplementation (50,000 IU/week) significantly increased serum IL-6, IL-1β, and IL-10 levels. These findings may refer to potential adverse effects during CS. High doses of VD_3_ significantly raised IL-6 levels, an important marker since an increase in its concentration is associated with an increase in the levels of CS. These findings confirmed that high or/and extensive doses of VD_3_ may potentially affect proinflammatory immune responses [21]. It has been demonstrated that 25OHD levels are lower in patients with many inflammatory diseases [22,23]. Further, inconclusive findings on the effects of VD_3_ supplementation for inflammatory conditions, including cytokines changes, were noted.

Results of the current trial were consistent with a prior RCT [24], showing that daily supplementation with 2000 IU of VD_3_ from baseline to 1 year had an 8% increase in IL-6 concentration in the intervention group compared with the placebo. Elevated IL-6 was also detected in children with multiple sclerosis who received VD_3_ [25]. Remarkably, the IL-10 findings of this trial were also consistent with other research reporting an elevation in IL-10 with no changes in IFN-γ levels in VD_3_-supplemented individuals [26]. Another study [27] also reported that after 6 months of VD3 supplementation, the levels of IL-6 were significantly elevated compared with baseline.

Previous clinical trials have typically been conducted under a treatment protocol close to or similar to our protocol, but they have been scarce. After extensive review, some clinical research studies were conducted under a protocol similar to this trial: four trials. In a trial conducted for 12 weeks on early chronic kidney disease [28], there were no changes in IL-6 levels. Conversely, dialysis patients, also studied for 12 weeks [29], showed a significant decrease in IL-6.

Patients with chronic renal impairment, such as hemodialysis patients, have elevated plasma IL-6 levels due to chronic inflammation and fluid overload. Reduced IL-6 clearance is noted with compromised kidney function, contributing to its retention. Therapeutic hemodialysis triggers inflammatory responses and increases IL-6 production [30,31].

The same dose of VD3 (50,000 IU per week) for 12 weeks lowered IL-6 levels in another RCT that aimed to examine the effect of VD3 and omega-3 fatty acid cosupplementation as an adjuvant chemotherapy [32].

It is important to note here that the observations of Al-Haidari and Khalighi were from trials conducted on patients under the influence of the treatment protocol for chronic diseases. In the Khalighi trial, all IBS patients received antispasmodic medication (Mebeverine, 135 mg twice daily) besides VD_3_ supplementation. Previous research has shown that the level of IL-6 in patients with diarrhea-predominant IBS was much greater than in healthy controls [33]. Therefore, the independent effect of VD_3_ supplementation on IL-6 has not been accurately evaluated.

Some studies have linked changes in the serum levels of IFN-γ and IL-10 observed after VD_3_ supplementation to the severity of VDD [34], suggesting that VD_3_ supplementation exerts the most influence on human immunity in the context of severe VDD. This is in contrast to many observational studies that support a potential inhibitory effect of VD_3_ supplementation on proinflammatory cytokines such as IL-6, IL-1β or/and TNF levels [35]. The effects of VD_3_ supplementation on human immunology have now been evaluated by large-scale RCTs that reported an absence of any effect of VD_3_ supplementation on IL-6 [36,37]. Notably, the majority of data are from Western countries. Ours is the first study to examine the effects of VD3 supplementation on the levels of CS-associated cytokines in the bloodstream of Jordanians with VDD. Similarly, VDD has been associated with elevated IL-6 levels [38], and VD_3_ downregulated IL-6 in some studies [39,40]. Contradictory results regarding IL-6 may be attributable to assessing the effects of VD3 supplementation on these cytokines in specific populations, multiple confounders, and discrepancies between research. These confounders include the duration and amount of VD3 supplementation, genetic background of patients, underlying clinical problems, impact of clinical therapy, and degree of VDD and insufficiency at baseline.

RCTs conducted on diabetic hemodialysis (HD) patients [41] or postmenopausal women without VDD [42] have revealed inconsistent results. Remarkably, results were quite different when smaller doses of VD_3_ over longer durations were used.

Considering the impact of a given dose in the different protocols, past clinical trials utilized different doses and durations of VD_3_ supplementation. Studies have shown that the effects of VD3 on reducing systemic inflammation may be greater in people who are overweight and have chronic inflammation and with more prolonged use [43,44]. Circulating IL-6 increases with age, BMI, and percentage of body fat mass. These factors such as being overweight with a slight elevation in serum leptin (approximately 8 ng/mL) and a mean age of around 40 years were detected in this trial. Nevertheless, stepwise regression did not show significant effects for these factors to be potential mediators in the association between 25OHD and IL-6 levels.

On the other hand, age and body weight factors are separately involved in the association between 25OHD and other proinflammatory cytokines (IL-1β and TNF). Elevated IL-6 and other cytokines observed in this trial contradict our previous hypothesis and are challenging to explain biologically. Considering whether the study sample is healthy people or patients, the effect of VD_3_ on those cytokines seems to be influenced by several factors, including baseline 25OHD levels and the dose, duration, and treatment protocol of VD_3_ [4,45]. The National Academy of Medicine of the U.S. deems a 600–800 IU VD daily intake adequate for most of the population. However, the U.S. Endocrine Society suggests daily 1500–2000 IU [46]. A total of 400 IU VD_3_ per day was suggested to treat individuals aged between 18 and 28 years [47]. This dose is approximately one-tenth the dose used in this trial, which is the most common treatment protocol in Jordan for patients with VDD. In the same context, 4000 IU VD_3_/day is the dose at which the risk of toxicity increases [48]. Therefore, a U-shaped association between serum 25OHD level and CVD risk has been proposed [49,50].

Further, the presence or absence of typical risk factors did not obscure the U-shape association [50]. Therefore, a U-shape association with extreme fluctuations in serum 25OHD levels may influence cytokine levels via its effects on the expression of their receptors. In this manner, we can explain the unexpected findings shown in this trial and previously [24]. Converse to previous studies and RCTs that reported the presence of a hypercytokiemia-reducing effect in VD3 therapy, Costenbader showed that an extensive VD_3_ dosage (2000 IU/day over 1 year) elevated 8% of IL-6 levels. According to these findings, high or/and extensive doses of these supplements, which are widespread in the community and a part of COVID-19 protocol treatment, require reconsideration, particularly during CS. Hence, it is improbable that these data can answer crucial issues about the possible effects of these supplements on the inflammatory pathway, even though they are frequently consumed by the general population [24]. Instead, it may induce a hypersensitive reaction accompanied by acute harmful consequences in people at risk of acute respiratory distress syndrome (ARDS), as observed in COVID-19 patients [51].

Although it has been established that VD3 supplementation reduces the incidence of influenza A [52], large amounts of IL-6 and IL-1β have been observed during CS [53]. There is new evidence that VDD is connected with higher levels of IL-6 in HIV patients [54]. There is currently no explanation for the variance in CS severity across COVID-19 patients. Accordingly, the results of this trial may point to a potential role for the sudden onset of 25OHD levels caused by high doses of VD_3_ supplements for this severity.

Another piece of evidence that came from a recent study showed that VD_3_ and IL-6 blockade (Tocilizumab) synergistically regulate rheumatoid arthritis by suppressing IL-17 [55]. Intriguingly, in the absence of serum VD3, the expression of IL-17A exhibited a positive feedback impact on the expression of IL-6. In contrast, under adequate conditions, IL-10 expression negatively impacted IL-17A and IL-6 expression; it raised the level of IL-10 mRNA expression in all groups. However, these effects were more pronounced in people with multiple sclerosis (MS). Eight weeks of treatment with 50,000 IU VD3 led to the downregulation of IL-6 and overexpression of IL-10 in 80% of MS patients [44]. Before this evidence, VD_3_ acted in synergy with Toll-like receptor (TLR) agonists and peptidoglycan (PGN) in inducing IL-6 and IL-10, whereas VD_3_ completely inhibited lipopolysaccharide (LPS) [55,56]. IL-6 and TGF-b may both have a role in developing Th17 cells that may play a vital function in antimicrobial immunity at mucosal barriers [57]. In response to the TLR activation of dendritic cells (DCs), it is well known that IL-6 blocks the inhibitory action of CD4, CD25, and regulatory T cells. It may interact with innate and adaptive immune responses [58].

Nevertheless, IL-6 decreases DC maturation and chemokine-receptor 7 expressions and may sometimes operate as an anti-inflammatory modulator [59]. Instead of exerting a general inhibitory impact on DCs, it has been suggested that VD3 promotes a delicate immunomodulation that inhibits adaptive immune responses while increasing innate immunological processes [60,61]. It is difficult to draw firm conclusions from this study due to its small sample size. Confirming these results, validating the reported cytokines as biomarkers of VD-mediated immune responses, and establishing the linkages between crucial immunological pathways and clinical outcomes all call for a larger clinical trial.

Contraindicating results might contribute to the varying doses and durations of VD_3_ supplements used in past trials. Therefore, based on the U-shaped curve, we hypothesize that high or extensive doses of VD3 may worsen serum cytokines associated with CS.

## 5. Conclusions

High doses of VD_3_ significantly raised IL-6 levels, which is an essential marker since elevated levels are linked to an increase in the severity of cytokine storms. Although the observations of this trial may refer to a potential negative effect of high-dose VD_3_ supplementation during a cytokine storm, careful implications are recommended, as this study did not investigate all cytokines involved in the cytokine storm. Accordingly, further trials are required to clarify the potential benefits of VD_3_ supplementation during a cytokine storm.

## Figures and Tables

**Table 1:** Frequencies and percentages of anthropometric and lifestyle variables at baseline (*n* = 75).

Variable	Category	Frequency	%
Group	Control	33	44.0
	VD_3_	42	56.0
	Total	75	100.0
Gender	Male	37	49.3
	Female	38	50.7
	Total	75	100.0
Morning sun exposure (20–30 min/day)	Yes	43	57.3
	No	32	42.7
	Total	75	100.0

Abbreviations: VD_3_, VD_3_ supplementation group.

**Table 2:** Statistical description of anthropometric parameters at baseline level (n = 75).

Parameter	Mean (SD)
Age (year)	38.37 (9.77)
Weight (kg)	78.51 (15.79)
Height (cm)	166.92 (7.42)
BMI (kg.m^−2^)	27.90 (4.76)
Waist (cm)	94.58 (14.12)
Hip (cm)	106.13 (11.52)
WHR	89.38 (11.39)

Abbreviations: SD, standard deviation; BMI, body mass index; WHR, waist/hip ratio.

**Table 3:** Descriptive summary of clinical variables at baseline (n = 75).

Parameter	Mean (SD)	Normal Range
25OHD (ng/mL)	17.29 (6.18)	30–50
PTH (pg/mL)	37.38 (7.57)	9–90
Ca (mg/dl)	9.30 (1.24)	8.6–10.3
PO_4_ (mg/dl)	4.05 (0.18)	2.5–4.5
Leptin (ng/mL)	7.86 (6.17)	NA

Abbreviations: 25OHD, 25 hydroxy vitamin D; PO_4_, phosphorus; NA, not applicable; SD, standard deviation.

**Table 4:** Levels of selected cytokines involved in the cytokine storm of COVID-19 at baseline in the entire study population with vitamin D deficiency (n = 75).

Parameter	Mean (SD)	Range *
IL-1β	3.24 (1.16)	0.17–24
TNF-α	32.38 (5.95)	0.93–26.8
IL-6	5.08 (5.16)	0.16–37.7
IL-10	2.09 (0.56)	0.01–19.8
(TNF-α/IL10)	1792.95 (1069.38)	
(IL-1β/IL10)	173.84 (97.53)	
(IL6/IL10)	243.21 (235.10)	

Abbreviations: IL-1β, interleukin-1 beta; IL-6, interleukin 6; IL-10, interleukin 10; TNF-α, tumor necrosis-alpha; SD, standard deviation. Note: * values are expressed as pg/mL—serum levels of cytokines for healthy people (age < 45 years) (23).

**Table 5:** Correlation of selected cytokine with baseline and follow-up 25OHD levels.

Variable	Baseline		Follow-Up	
	R	*p*-Value	R	*p*-Value
TNF-α	−0.072	0.538	−0.027	0.867
IL1	−0.280	0.015	0.215	0.171
IL6	−0.174	0.136	0.037	0.815
IL10	−0.206	0.076	−0.059	0.712

Note: R, correlation coefficient.

**Table 6:** Changes in the serum levels of 25OHD and PTH.

Variable	Group	Control	D_3_	*p*-Value
25OHD	Baseline	18.42 ± 7.36	16.41 ± 4.99	P^B^ = 0.163
	Follow-up	17.31 ± 6.74	41.39 ± 12.19	P^C^ < 0.001
	Change	−1.11	24.98	
	P^A^	0.062	<0.001	
PTH	Baseline	36.75 ± 8.49	37.88 ± 6.82	P^B^ = 0.524
	Follow-up	33.85 ± 10.62	16.69 ± 8.72	P^C^ < 0.001
	Change	−2.90	−21.19	
	P^A^	0.052	<0.001	

Abbreviations: P^A^, *p*-value for paired sample *t*-test; P^B^, *p*-value for two independent sample *t*-tests at baseline; P^C^, *p*-value for two independent sample *t*-test at follow-up; CV%, the coefficient of variation; D_3_, vitamin D3 supplementation group; PTH, para thyroid hormone; 25OHD, 25hydroxy vitamin d.

**Table 7:** Changes in the serum levels of selected cytokines associated with cytokine storm at baseline and 10-week follow-up.

Variable	Group	Control	D_3_	*p*-Value
IL-1β (pg/mL)	Baseline	3.27 ± 1.37	3.22 ± 0.99	P^B^ = 0.852
	Follow-up	3.59 ± 2.71	7.63 ± 2.36	P^C^ < 0.001
	Change	0.32	4.41	
	P^A^	0.574	<0.001	
IL-6 (pg/mL)	Baseline	4.55 ± 2.62	5.5 ± 6.51	P^B^ = 0.431
	Follow-up	4.85 ± 4.84	26.99 ± 14.47	P^C^ < 0.001
	Change	0.30	21.49	
	P^A^	0.750	<0.001	
TNF-α (pg/mL)	Baseline	31.16 ± 5.22	33.34 ± 6.36	P^B^ = 0.116
	Follow-up	33.65 ± 5.15	33.50 ± 6.18	P^C^ = 0.910
	Change	2.49	0.16	
	P^A^	0.100	0.899	
IL-10 (pg/mL)	Baseline	2.20 ± 0.52	2.01 ± 0.59	P^B^ = 0.159
	Follow-up	2.39 ± 1.39	4.46 ± 4.67	P^C^ = 0.016
	Change	0.20	2.45	
	P^A^	0.433	0.001	

Abbreviations: P^A^, *p*-value for paired sample *t*-test; P^B^, *p*-value for two independent sample *t*-test at baseline; P^C^, *p*-value for two independent sample *t*-test at follow-up of trial.

**Table 8:** Significant correlations of cytokine and ratio levels with trial variables at follow-up.

Dependent Variable	Univariate Effect Estimate	Coefficient				
		B	F	R	R^2^	*p*-Value
TNF-α	Age	0.257	8.220	0.413	0.170	0.007
IL-1β	Weight	0.053	4.294	0.311	0.097	0.045
TNF-α /IL10	WHR	0.348	5.200	0.348	0.121	0.024

Abbreviations: WHR, waist/hip ratio.

## Data Availability

Not applicable.
